# Parkinson rehabilitation in nursing homes: a qualitative exploration of the experiences of patients and caregivers

**DOI:** 10.1007/s41999-022-00647-z

**Published:** 2022-05-11

**Authors:** Hester Fidder, Joannina J. Jaski, Eskeline Elbertse, Anouk M. van Loon, Annelie A. Monnier, Marike E. de Boer, Aafke J. de Groot

**Affiliations:** 1 Department of Medicine for Older People, Amsterdam UMC, Location Vrije Universiteit Amsterdam, de Boelelaan 1117, Amsterdam, The Netherlands; 2 Amsterdam Public Health, Aging and Later Life, Amsterdam, The Netherlands; 3 Vivium Naarderheem Geriatric Rehabilitaton Center, Naarden, The Netherlands; 4 Beweging 3.0, Geriatric Rehabilitation Center De Pol, Nijkerk, The Netherlands

**Keywords:** Parkinson disease, Parkinsonian disorders, Rehabilitation, Parkinson disease*/rehabilitation, Skilled nursing facility, Nursing home, aged, Qualitative research

## Abstract

**Purpose:**

Worldwide, an increasing number of people are diagnosed with atypical Parkinsonism or idiopathic Parkinson’s disease (PD). Periods of acute functional decline, triggered by acute disease, are common. Rehabilitation is often necessary to restore functioning. Skilled nursing facilities (SNFs) in the Netherlands have developed evidence-based geriatric rehabilitation for Parkinson (GR-P) programs. However, data on the experiences and needs of patients and their caregivers are lacking. This study aims to address these, in order to propose recommendations for improvement.

**Methods:**

We performed a qualitative study, using semi-structured interviews in two Dutch SNFs offering GR-P. Nine patients with PD and six informal caregivers were included. We subjected verbatim transcripts of 15 interviews to qualitative analysis.

**Results:**

Data saturation was reached after 15 interviews. Three overarching themes emerged: (1) autonomy, (2) sharing information and (3) contact with others. Loss of autonomy was linked to the underlying disease and the rehabilitation environment itself. Patients and caregivers felt overwhelmed by events before and during rehabilitation, expressing a need to receive information and discuss prior experiences. They considered communication between hospitals and SNFs to be poor. Patients did not always appreciate contact with peers. Both patients and caregivers appreciated empathic healthcare personnel with a firm knowledge on PD.

**Conclusions:**

Autonomy, sharing information and contact with others are central themes for patients and caregivers during GR-P in SNFs. We recommend actively exploring these three central themes with every patient and caregiver entering a GR-P program and offering staff continuing education on PD, in order to improve care.

## Key summary points

**Aim:**

What are experiences and needs of patients with parkinsonism or idiopathic Parkinson’s disease who participate in geriatric Parkinson rehabilitation programs in skilled nursing facilities?

**Findings:**

Autonomy, sharing information and contact with others are central themes for patients and caregivers during Parkinson rehabilitation.

**Message:**

To improve care, we recommend actively exploring these three central themes with every patient and caregiver entering a Parkinson rehabilitation program, as well as offering staff continuing education on the disease.

## Introduction

Atypical parkinsonism and idiopathic Parkinson’s disease (collectively denoted in the following as PD, or specified as such where relevant) refer to a group of progressive neurodegenerative diseases primarily known for their effect on the extrapyramidal system. In 2016, it was estimated that 6.1 million individuals worldwide had PD, a figure more than double that of 1990. A relatively conservative estimate predicts that a doubling of the number of cases over the next 30 years would yield more than 12 million people with PD worldwide by about 2050 [1]. People suffering from PD characteristically display motor symptoms such as rigidity, bradykinesia, hypokinesia, resting tremor and postural instability. In addition, important non-motor symptoms such as autonomic dysfunction and neuropsychiatric symptoms exist [2]. Progression of motor symptoms may lead to complications such as malnutrition and worsening of physical condition, causing adverse events and hospital admission [3].

In the Netherlands, community-dwelling, frail people with complex geriatric morbidity like PD, who suffer from an episode of acute or sub-acute functional decline, are eligible for admission to geriatric rehabilitation wards of a skilled nursing facility (SNF). SNFs offer integrated multidisciplinary treatment and care. Generally, patients follow a 4–6-weeks medium-intensive geriatric rehabilitation program aimed at restoring their functional abilities or adapting to new disabilities, thus stimulating societal participation and facilitating living at home again.

There is some evidence of positive effects of geriatric rehabilitation in older patients with PD. One study demonstrated that short (< 6 weeks) intensive multidisciplinary rehabilitation in a SNF with a dedicated rehabilitation program significantly improved cognitive function and postponed admission to chronic care [4]. SNFs have developed specialized geriatric rehabilitation programs for Parkinson (GR-P) accordingly, for patients with PD as a primary cause of functional decline or as a comorbidity accompanying other causes of acute deterioration. Most patients with PD admitted to a SNF are in advanced stages of the disease, characterized by multi-domain problems. Speech, other motor problems and neuropsychiatric symptoms often limit communication. Therefore, caregivers play an essential role in the rehabilitation process [5]. They provide important information on previous health problems and home situation. Staff relies on their information about issues like motor symptoms, behavioural approaches and medication habits. In rehabilitation programs, caregivers are invited to be present at therapy sessions and participate in treatment decisions, helping both staff and patient to put results into perspective. Finally, the caregiver must be informed about, and supported in, continuing care at home after discharge. During rehabilitation, caregivers are, thus, actively involved.

Reports on expectations, needs and experiences of older people with PD and their caregivers concerning rehabilitation are scarce. This study aims to explore their perspective, in order to make recommendations on tailoring of GR-P programs to their specific needs and wishes.

## Materials and methods

### Design

This report was composed in accordance with the consolidated criteria for reporting qualitative research (COREQ) [6], (see Table 1). To explore the experienced reality of patients and caregivers, we used a pragmatic approach [7] and obtained data via individual semi-structured interviews with patients with PD admitted for geriatric rehabilitation in SNFs and their caregivers. To minimize the influence of the personal opinions of interviewers on the answers of patients and caregivers, interviews were conducted by three researchers who were uniformly trained, made use of the same topic list and were not involved in participants’ rehabilitation nor employed at the SNFs where interviews took place.

### Setting

This study was performed in two SNFs in the Netherlands, both specializing in GR-P. One facility is a suburban geriatric rehabilitation center with a total of 120 beds, the other is a 34 beds geriatric rehabilitation department of a nursing home in a rural area. These facilities provide rehabilitation-oriented care based on the protocols of the Dutch national Parkinson Guidelines. The multidisciplinary team in each facility consist of experienced professionals, including nursing staff and paramedics (a physio therapist, occupational therapist, speech therapist, dietitian and psychologist), acting under the medical supervision of an attending physician. All professionals are members of and trained by ParkinsonNet [8]. Each year, in each facility, approximately 15–20 patients are admitted for GR-P after acute functional decline related to progression of PD itself or an intercurrent disease or trauma. The mean duration of stay in both facilities is 4–6 weeks.

### Participants

All patients with PD admitted for rehabilitation and their primary caregivers (as proxy-informants) were eligible for participation in our study. We used convenience sampling, there were no assumptions made beforehand that patient characteristics would influence results. Patients were asked to identify their primary caregiver but having no caregiver did not prevent patient participation. The attending physicians invited both patients and caregivers to meet face-to-face approximately 3–5 weeks after admission and provided them with verbal and written information about the study. None of the invitees declined to participate. The only exclusion criterion was an inability to give informed consent because of cognitive or communication problems. Recruitment of participants continued until data saturation was reached. A total of nine patients were included, of which four lacked an informal caregiver to be interviewed. Six caregivers were included, of whom one had a partner that could not be included.

### Data collection

Semi-structured interviews based on a topic list (see Table 2) were performed between February and August 2020. The topic list was developed in consensus with the research team and with rehabilitation professionals. Main topics were admission to geriatric rehabilitation, being a (temporary) resident, goal setting for the rehabilitation treatment, nursing and medical care and outcome of rehabilitation. The topic lists were finalized based on feedback from the interviewers after the first (pilot) interviews. All interviews were audio recorded and took about 45 min–one hour. Field notes were made after interviews. In addition, basic demographic and clinical characteristics of participants were extracted from medical records (patients) or collected at the start of the interview (informal caregivers). The interviews were conducted by three independent healthcare professionals who had received instruction and had no personal or professional relationship with study participants. All interviews were performed individually and face-to-face in the participating SNFs or at patients’ homes, except for three interviews that were performed via telephone, because of restrictions due to the COVID-19 pandemic. No non-participants were present during interviews. Inclusion of patients stopped when saturation was reached (i.e., when no new or interesting insights or points of view that seemed relevant for this study were expressed).

### Data analysis

All interviews were transcribed verbatim and subjected to thematic content analysis [by HF, AG, JJ], applying the Gale et al. Framework Method, a tool for thematic qualitative content analysis, that provides a systematic model for managing and mapping data [9]. Analysis incorporated a cyclical and iterative process of reading and re-reading the transcripts in order to code the text and identify themes. First, meaningful words or passages were coded, i.e., labeled with names or simple phrases expressing meaning. There was triangulation of researchers, with three researchers performing interviews and data being analyzed independently: the first four transcripts of patients’ interviews were also coded independently by two other team members [AG and JJ]; discrepancies were discussed and modified when necessary. After that, the whole transcript was re-read and codes were compared, re-labeled and thereafter sorted into categories. Consensus on codes and categories was ensured through peer debriefing within the research team. A codebook was developed and applied to patient and caregiver interviews. Before starting the analysis process, we had decided to analyze the interviews of caregivers and patients jointly, because we expected no significant differences, as caregivers had been asked to answer questions from patient’s perspective (proxy- perspective). The use of the framework helped conduct the thematic analysis by enabling comparisons within and between cases. Deviant cases were described and the corresponding quotations are given in the article. The data were organized into the identified themes and discussed within the research team for further interpretation and verification of findings. Data analysis was supported by qualitative data software (Atlas.ti 8.4, Scientific Software Development, GmbH, Berlin, Germany). No direct member check was performed, but during the interview oral summaries were made by the interviewer to check whether information had been understood correctly.

### Ethics

Informed consent was obtained from all participants. The medical ethics review committee of Amsterdam University Medical Center (METC) approved the study protocol (METC file no. 2019.533). The interviewers were not employed in any of the participating SNFs and had no interest in any particular result of the interviews; the only goal was to explore experiences of participants.

## Results

A total of 15 interviews were conducted with patients (*n* = 9) and informal caregivers (*n* = 6). Table 3 shows demographic and clinical characteristics.

As the process of analysis revealed no substantial or relevant differences between patients’ and caregivers’ perspectives; results account for all respondents and no subgroup results are presented. Also, there were no important differences between the two included SNFs.

Three overarching themes emerged from the data: (1) autonomy, (2) sharing information, and (3) contact with others. Ten subthemes were identified. Themes and subthemes are shown in Table 4.

### Theme 1. Autonomy

Autonomy was a theme found in all interviews with patients and caregivers. Four subthemes within this theme were: (1) dependence on help and having to wait, (2) supervision during rehabilitation, (3) accessibility of the physical environment and (4) regaining independence during rehabilitation (Table 4).

#### Subtheme 1.1 Dependence on help and having to wait

Generally, patients feared deterioration and the loss of autonomy accompanying the progressive nature of their disease. Admission to a geriatric rehabilitation ward was associated with having to wait for help and a (further) reduction of independence. Patients noted that immediate assistance of nursing staff was not always available for unplanned and often urgent basic activities of daily living. A caregiver described how this made his wife feel:

She really hated the fact that she always had to wait, she always told me that. How would you feel about that, when people see you as a worthless person? “Yes, you’ll have to wait for a while madam, we don’t have time for that.” (…). And that’s the problem, you see, it makes you feel so small. That’s terrible. It makes you feel so insecure. [Caregiver 6]

Patients were found to be reluctant to hand over control of their Parkinson medication, as described by this caregiver of a patient with pump-delivered levodopa therapy:

It’s very annoying, also for my wife. She was not very keen on getting that [medication] pump, and she has tried to prevent it for as long as she could. And she said: “I’ll not be in charge anymore, because if I get that pump, it’ll take over from me, and that’s fine, but I’ll not be able to regulate it myself anymore”.

[Caregiver 1]

Less dependent patients or their caregivers did not experience a loss of autonomy:

No, no, I don’t notice that he has to wait. Because, you see, he is still able to do many things independently. [Caregiver 5]

#### Subtheme 1.2 Supervision during rehabilitation

To reduce the risk of adverse events, patients were often advised to perform activities under supervision until they could act alone safely. Some participants rejected this advice:

Yes, I’ve fallen two times. But then I think, there are so many people who fall every now and then. They are overprotective here, and that’s very sweet of course. But, I say, I don’t want to spend the rest of my life locked up in a room. [Patient 6]

Due to safety regulations, medication is initially (and often permanently) distributed by nursing staff and intake is supervised. Some patients reported medication being administered too late, or objected to supervised medication administration:

And that method of controlling things, that I had come up with myself, that method has been taken over, partially or completely. It has to do with the possibility of personally controlling your own packaged medication roll and checking if everything is still correct, the way it had been before. And that’s the biggest problem. They give you a cup [with the medication] and tell you: “All right? Now swallow it.” [Patient 2]

#### Subtheme 1.3 Accessibility of the physical environment

Patients also reported barriers in the physical rehabilitation environment, such as small remote-control buttons and missing support brackets in toilets. This increased their dependence on others even further.

You have to push this button to open the door (…) they are a bit small, and you have to push hard. And my thumb doesn’t work so well. [Patient 8]

Seemingly small issues such as these may negatively affect patient autonomy, by increasing dependence on others.

#### Subtheme 1.4 Regaining independence during rehabilitation

As a result of rehabilitation, all participants noticed progress in their functioning and they became less dependent. Patients assessed progress particularly by their ability to walk. Regaining prior functional level was a positive experience:

Instead of shuffling, I am now able to take proper steps… (…) a very big jump forward. And I find the way in which it happens wonderful too. You feel as if you go from being a child to becoming yourself again. [Patient 2]

### Theme 2. Sharing information

A second overarching theme that emerged was sharing information. Three subthemes within this theme were: (1) a desire to share personal stories, (2) making sense of the rehabilitation environment and (3) interprofessional communication.

#### Subtheme 2.1 A desire to share personal stories

Many patients and caregivers had gone through a difficult period before their admission to rehabilitative care. Participants often expressed a desire to talk about their prior experiences. They said it was important that therapists were fully aware of the events and situation before admission:

I’ve taken care of her for 24 hours a day, from December onwards, because she could not lift her trousers anymore, nor wipe her buttocks or go to bed by herself… And then, all that pain... And I want to tell you this, so that you know what has happened to us before, because that has been important during these weeks. At least, for us as persons, it has been very important. [Caregiver 6]

Patients primarily seemed eager to be heard, to tell their story and share their experiences. However, many declined professional counseling.

#### Subtheme 2.2 Making sense of the rehabilitation environment

Many patients had difficulty to grasp their situation. Several basic disease-related topics (such as diagnosis, medication) and rehabilitation-related topics (such as reason for admission, rehabilitation goals) were unclear to them, and they wished to receive more information on this subject. Some struggled with organizational issues, such as the identity and function of the various professionals. For instance, different uniform colors of staff members were confusing a patient who tried to give a (non-existent) meaning to this phenomenon, as is illustrated by her comment: “A blue vest, you think, she must be in charge then” [patient 8].

To another patient, the goal of the assignment provided by the occupational therapist was unclear:

Then we enter the room and she says, I want you to make me some coffee. And I think, what’s going on here, making coffee? Looking back, I understand, but not at first (...). Now I understand the intention, it was just to see if everything still functions properly upstairs. [Patient 1]

Especially early in rehabilitation, patients said they felt overwhelmed because so much was going on. Some patients indicated that it had taken weeks before they understood what the purpose of the rehabilitation was.

Only now during the last weeks, I’m starting to understand why I’m here. It’s not only about medication, which I thought was the problem, but it’s about more than that. [Patient 2]

Some patients indicated that they were not notified when medication was changed and wished to be informed on this subject. Other patients noted inadequate information supply on group activities. Caregivers generally expressed a desire of being better informed about the situation of their loved ones. One caregiver suggested offering an information leaflet at admission followed by an appointment after approximately two weeks. One (comparatively young) caregiver appreciated the option of using the electronic patient record as a way of keeping informed about her mother’s situation.

#### Subtheme 2.3 Interprofessional communication

Patients and caregivers noted sub-optimal communication between professionals of different healthcare institutions (such as SNF, pharmacy, hospital or general practice), causing problems like medication errors. In addition, patients and caregivers described how they themselves had had a hard time contacting or receiving proper information from health care professionals in the past. For instance, one caregiver described how ill-informed she felt about her husband’s deep brain stimulation (DBS) implantation:

But the information from the hospital was very unclear. I thought, he’ll have the operation in June, they’ll adjust it, and then it’s done. And then afterwards we heard that the adjustment could take four to six months (…). I: And how does it feel, to hear that information afterwards? C: Just terrible… It’s such a deception. [Caregiver 5]

### Theme 3. Contact with others

Contact with others emerged as a third major theme. Subthemes are: (1) positive and negative experiences with peer contact, (2) staff members showing empathy and (3) valuing expertise of staff.

#### Subtheme 3.1 Positive and negative experiences with peer contact

Contact with peers brought about both positive and/or negative experiences in patients. Positive aspects were for instance the social aspects of dining together in the restaurant, or, as one relatively young patient described, “I like being able to help other, older peers” [patient 4]. Exercising with fellow patients was also highly valued and participants wished to do this more often. Negative experiences were described by a patient who did not enjoy the constant presence of other patients with PD, which lead to expressions as: “I don’t need to be surrounded by Parkinson all the time” [patient 3]. Other participants felt left out, lonely or were bothered by others observing them:

They notice everything, these people (…). Last night too, someone said: madam, you are wriggling so much! Doesn’t it tire you? (..)

I: Does it bother you, when you hear things like this?

P: Yes, sometimes… [Patient 7]

#### Subtheme 3.2 Staff members showing empathy

All patients were satisfied with the way they were treated by staff members, valuing their concern and friendly attitude. Patients and caregivers also appreciated it when staff members showed their own emotions. One caregiver remarked:

Well, last time I was talking to the doctor, she noticed me getting emotional, and it touched her. And that made me think: “Look, she’s a human being too. (..) She has a human side and she understands me.” [Caregiver 3]

#### Subtheme 3.3 Valuing expertise of staff

Patients and caregivers predominantly felt that all staff members had enough expertise and valued this. Furthermore, caregivers appreciated learning new illness-related information. For instance, one caregiver [caregiver 3] explained how he had learned that doing multiple different things at the same time was too challenging for his wife and how this knowledge helped him in the daily care for her.

However, patients who received duodenal levodopa infusion stated that nursing staff was not always capable in handling the pump. Consequently, these patients did not fully trust staff’s expertise in this field. A patient remarked:

Sometimes they ask me what to do [with the pump]. And I’d like to do it myself, but I’m not able to in the morning. (...) In the morning, I can’t talk well yet, I can’t explain things correctly. And then they ask me: ‘Excuse me madam, what are you saying?’ That is rather tiring, in the mornings. [Patient 7]

There were no differences between wishes and needs of patients and caregivers; the same themes emerged in both groups.

## Discussion

This study shows that issues related to autonomy, communication and contact with others are important for patients with PD and their informal caregivers during GR-P in SNFs. We observed no differences between patients and caregivers, and between patients with and without a caregiver. To our knowledge, this is the first study exploring experiences and needs of both patients with PD and their informal caregivers during GR-P. In the next section, we will compare our findings with results of studies that resemble ours [10–13].

Many patients experienced a loss of autonomy during rehabilitation, as indicated by both patient and caregivers. This feeling was linked to the underlying disease, but also related to the rehabilitation environment itself. Their physical deterioration had led to an increased dependency on nursing staff and having to wait for help with basic activities of daily living (ADL). Staff advice, aimed at minimizing the risk of falling or medication safety increased dependency even further. Problems with administration of medication, and especially with medication being administered too late, represented an important issue for patients and caregivers. Patients wish to regain control and maintain autonomy and all participants indeed noted functional progress during rehabilitation.

Our results are in line with other studies concerning idiopathic Parkinson’s disease (IPD) patients [10, 14, 15]. A multidisciplinary in-patient rehabilitation program for patients with IPD was found to contribute to a rediscovered sense of autonomy [10]. Another study showed that having IPD undermines patients’ self-efficacy and autonomy by making them dependent on others for often simple tasks [15]. Furthermore, the value of autonomy for patients with PD has been investigated in home-dwelling patients; more self-management was amongst their greatest wishes [14].

Moreover, several rehabilitation studies report on the value of autonomy during general or disease-specific rehabilitation. Autonomy was found to be essential in client-centered rehabilitation and a pre-requisite for effective participation [16]. A qualitative study assessing COPD patients’ perceptions of an in-patient pulmonary rehabilitation program [11] described how people experienced a restriction of freedom in the first weeks of rehabilitation; respecting autonomy helped to create a more autonomous motivation and patients developed a desire to self-regulate their lives again. The authors therefore advised healthcare professionals to apply an autonomy-supportive counseling style. Finally, a study focusing on stroke rehabilitation, described how the inability to exercise choice negatively influenced recovery. Fostering autonomy was recommended to improve rehabilitation for stroke patients [12].

According to our results, patients desired to share their personal stories. A review on storytelling interventions for chronic disease self-management [17] concluded that storytelling has the potential to provide patients with a more active role in their health care, but measures of impact differed across interventions because of the differences between the diseases. Furthermore, patients struggled to make sense of the rehabilitation environment. It remained unclear whether they did not receive adequate information (as some stated) or had difficulty comprehending it. Many patients at some stage felt uninformed about disease and rehabilitation-related topics, such as diagnosis, medication, reason for admission and rehabilitation goals. Some had difficulties with organizational issues. Although on admission patients often felt overwhelmed, they had no specific remarks on the admission process itself. Caregivers noted and regretted poor interprofessional communication, especially regarding medication. During the rehabilitation period, they discovered that their knowledge on the disease and the rehabilitation environment increased.

These findings correspond with the results of two qualitative studies investigating needs of patients with IPD at home or in a chronic care facility. Patients were found to wish for more education on symptoms, treatment options and coping strategies for themselves, their families and health care professionals [17, 18] and better interdisciplinary collaboration [18]. For stroke patients undergoing rehabilitation, accurate information and communication between participants and therapists was essential to a good rehabilitation experience; poor communication was disempowering and had the potential to diminish autonomy, confidence, and motivation [12].

In our study, patients appreciated the empathic way they were approached by staff during rehabilitation. Peer contact was experienced either positively or negatively. Some valued it, for instance when finding support, being able to help others, or when looking for distraction. Negative experiences included embarrassment over or fear of visibility of motor symptoms to others during off-periods and the constant confrontation with their disease.

The same ambiguity towards peer contact was noted in two publications on home-dwelling, hospitalized or nursing home persons with IPD, who reported feelings of anxiety, embarrassment and fear (e.g., for public motor symptoms) [15, 18]. Shame was found to be another often-unrecognized emotion in people with IPD [19]. A reluctance to be confronted with other patients and the (future) severity of the disease also occurs [15, 20]. On the other hand, some patients accept the progressive nature of their disease [15]. In all three publications, peer support was described a valuable resource to some [15, 18–20].

Regarding in-patient rehabilitation for patients with other chronic conditions, a study on patients with Huntington disease (HD) found that participants highlighted being a member of an ‘HD-group’ as a valuable experience, despite tensions and conflicts [21]. For patients with multiple sclerosis undergoing out-patient rehabilitation, peer group discussions were judged to be helpful by nearly all participants. Authors concluded that the importance of peers and peer support should be considered in rehabilitation planning and related recommendations [13].

Furthermore, a meta-analysis concluded that patients identified the importance of emotional support in feeling valued, accepted, and not judged or discriminated because of their IPD [22]. Patients with IPD were found to explicitly feel a need for more emotional support and empathy [20].

We found that patients and caregivers generally felt that staff members had enough Parkinson-specific knowledge. The SNFs participating in our study re known to be specialized Parkinson rehabilitation centers, which may have caused patients to positively value the knowledge and practical skills of personnel. Both facilities have been delivering GR-P to 15–20 patients per year each for several years, and their nurses, therapists and physicians follow frequent and recurrent education as is recommended [20]. Hence, healthcare workers in our study might have had a better understanding of Parkinson-specific issues than those not working with this specific patient group.

There are a few strengths and limitations to be addressed with regard to our study. Some patients and caregivers showed signs of reduced cognitive functioning during the interviews. However, this does not make their needs and wishes less relevant. Cognitive impairment is a common non-motor symptom of patients with PD admitted for geriatric rehabilitation. Although some participants lingered on certain questions or deviated from the subject, none of their comments were inconsistent or incomprehensible for the researchers.

Regional health care factors may have influenced patients’ experiences. However, themes did not differ between the two centers. Also, the results of our study are in line with other qualitative studies with patients with PD and their caregivers and correspond with international data on rehabilitation experiences of patients with other chronic diseases [11–13, 21]. This suggests that our findings can be applicable to other SNFs who offer rehabilitation for patients with PD. Even though the specificity of the setting and type of care may limit generalization of our results to other settings, our findings do agree with studies investigating patient perspectives on living with chronic illness in adults [23].

Although data were collected by three different interviewers with differences in age, sex and professional background, the risk of bias was reduced through the use of a uniform instruction and a topic list for guidance. In addition, analysis was performed independently by other researchers of the team. Analyzing patients and caregivers’ results jointly was done because patients and caregivers were both considered to be important sources of information on the rehabilitation experience. This notion is a very common observation in rehabilitation care in the Netherlands. It is based on experiences in daily practice, where caregivers are closely involved with patients during the rehabilitation process. Thus, we aimed at also exploring caregivers’ views and opinions. Unfortunately, not all patients had informal caregivers to interview. However, this too is a common situation in GR-P. We found no differences in themes between patients with or without a caregiver.

In conclusion, this study provides new insights into the rehabilitation experiences of older patients with PD and their caregivers. The themes that emerged are currently not incorporated in the Dutch GR-P programs of SNFs. We, therefore, recommend that GR-P programs in SNFs should be tailored to the needs of patients with PD and their informal caregivers taking into account the three central themes found in this study.

Staff should make efforts to warrant and endorse patient autonomy, by engaging patients and caregivers in decisions affecting their independence and accepting that patients may want to accept certain risks to maintain autonomy. In addition, actively inquiring after potential practical barriers in the physical environment may help staff to reduce limitations and patient dependence. It is important to actively and repeatedly explore and address individual information needs of patients and caregivers. Also, the need to share personal stories should be addressed, either by regular staff, or by experienced volunteers or peers.

Contact with peers must be offered, but staff should allow patients to decline participation when preferred. Group therapy is appreciated and should therefore be part of the rehabilitation plan. Regular evaluations of these activities are advised.

Patients and caregivers appreciated empathic health care personnel with robust knowledge. This underlines the importance of education and practical experience for health care workers dealing with people with PD.

Caregivers should be actively included during rehabilitation as they are important informers, and they guard the continuity of care for their loved ones with PD during and after rehabilitation.

Further research should evaluate the effect of these recommendations on patient and caregiver experiences during geriatric rehabilitation for PD in SNFs and investigate the presence of similar overarching themes in other treatment settings.

## Appendix (see Tables 1, 2, 3, and 4)

**Table 1:** Consolidated criteria for reporting qualitative studies (COREQ): 32-item checklist^a^

No. item	Guide questions/description		Reported on page #
Domain 1: Research team and reflexivity			
Personal Characteristics			
1. Inter viewer/facilitator	Which author/s conducted the interviews or focus group?	Three health care professionals conducted the interviews. Their characteristics are described in Table 3 (p.19)	Table 3
2. Credentials	What were the researcher’s credentials? E.g., PhD, MD	EE, HF, JJ, AG: MD MdB, AvL, AM: PhD	1
3. Occupation	What was their occupation at the time of the study?	EE, HF, JJ, AG: MD MdB, AvL, AM: senior researchers	1
4. Gender	Was the researcher male or female?	Researchers: 7 females Interviewers: 2 females, 1 male	Not reported
5. Experience and training	What experience or training did the researcher have?	All interviewers received training as how to perform interviews	5
Relationship with participants			
6. Relationship established	Was a relationship established prior to study commencement?	The researchers were not known to the participants prior to the study	5
7. Participant knowledge of the interviewer	What did the participants know about the researcher? e.g., personal goals, reasons for doing the research	Researchers told participants that their goals were to contribute to more knowledge on the study subject, and that they had no personal interest in any specific outcome. They were also told that results might be published in an international publication and would be informed about the proceedings	Not reported
8. Interviewer characteristics	What characteristics were reported about the inter viewer/facilitator? e.g., Bias, assumptions, reasons and interests in the research topic	Demographic characteristics of interviewers (age, sex, professional background) are described in Table 3 To minimize the influence of the personal opinions of interviewers on the answers of patients and caregivers, interviews were conducted by three researchers who were not involved in participants’ rehabilitation nor employed at the SNFs where interviews took place Although data were collected by three different interviewers with a variety in age, sex and professional background, the risk of bias was reduced through the use of a uniform instruction and a topic list for guidance. In addition, analysis was performed independently by other researchers of the team	4, 14, 19
Domain 2: study design			
Theoretical framework			
9. Methodological orientation and Theory	What methodological orientation was stated to underpin the study? e.g., grounded theory, discourse analysis, ethnography, phenomenology, content analysis	To explore the experienced reality of patients and caregivers we used a pragmatic approach. Within this pragmatic approach we used a combination of analytic strategies and procedures, which were already further explained under the heading ‘Data analysis’ in our manuscript (p. 5)	4, 5
Participant selection			
10. Sampling	How were participants selected? e.g., purposive, convenience, consecutive, snowball	We used convenience sampling	4
11. Method of approach	How were participants approached? e.g., face-to-face, telephone, mail, email	The attending physicians invited both patients and caregivers face-to-face and supplied them with written information	4
12. Sample size	How many participants were in the study?	9 patients and 6 caregivers were included in this study	6
13. Non-participation	How many people refused to participate or dropped out? Reasons?	None of the invitees declined to participate	5
Setting			
14. Setting of data collection	Where was the data collected? e.g., home, clinic, workplace	All interviews were performed face-to-face in the participating SNFs or at patients’ homes, except for three interviews that were performed via telephone, because of restrictions due to the COVID-19 pandemic	5
15. Presence of non-participants	Was anyone else present besides the participants and researchers?	No non-participants were present during interviews	5
16. Description of sample	What are the important characteristics of the sample? e.g., demographic data, date	Table 3 (appendix) shows demographic and clinical characteristics of participants	Appendix p. 1
Data collection			
17. Interview guide	Were questions, prompts, guides provided by the authors? Was it pilot tested?	The topic list was developed in consensus with the research team. Main topics were admission to geriatric rehabilitation, being a resident on a ward, goal setting for the rehabilitation treatment, nursing and medical care, attitude of staff and outcome of rehabilitation. Two pilot interviews were performed, after which the topic lists were altered based on feedback from the interviewers	5
18. Repeat interviews	Were repeat interviews carried out? If yes, how many?	No repeat interviews were carried out	N/A
19. Audio/visual recording	Did the research use audio or visual recording to collect the data?	All interviews were audio recorded	5
20. Field notes	Were field notes made during and/or after the interviews or focus group?	Field notes were made after interviews and meetings with the research team	5
21. Duration	What was the duration of the inter views or focus group?	All interviews took about 45 min to one hour	5
22. Data saturation	Was data saturation discussed?	Inclusion of patients stopped when saturation was reached (i.e., when no new or interesting insights or points of view that seemed relevant for this study were expressed)	5
23. Transcripts returned	Were transcripts returned to participants for comment and/or correction?	No direct member check was performed	N/A
Domain 3: analysis and findings			
Data analysis			
24. Number of data coders	How many data coders coded the data?	The first four transcripts of patients’ interviews were also coded independently by two team members [AG and JJ]; discrepancies were discussed and modified when necessary	5
25. Description of the coding tree	Did authors provide a description of the coding tree?	A coding tree description is not given	Not reported
26. Derivation of themes	Were themes identified in advance or derived from the data?	Themes were not identified in advance. Three main themes were derived from data	6
27. Software	What software, if applicable, was used to manage the data?	Atlas.ti 8.4 was used to manage the data	6
28. Participant checking	Did participants provide feedback on the findings?	No, although indirect member checks (summarizing and checking whether the interviewee had been understood correctly) were performed during the interviews	6
Reporting			
29. Quotations presented	Were participant quotations presented to illustrate the themes/findings? Was each quotation identified? e.g., participant number	Please see the results section of the manuscript	6–12
30. Data and findings consistent	Was there consistency between the data presented and the findings?	There was consistency between the data presented (main themes) and findings	6–12
31. Clarity of major themes	Were major themes clearly presented in the findings?	Major themes were presented in the results section and in Table 4 (appendix)	6–12, appendix p. 20
32. Clarity of minor themes	Is there a description of diverse cases or discussion of minor themes?	Deviant cases were described and the corresponding quotations are given in the article	6–12

International Journal for Quality in Health Care. 2007. Volume 19, Number 6: pp. 349–357

^a^Developed from: Tong A, Sainsbury P, Craig J. Consolidated criteria for reporting qualitative research (COREQ): a 32-item checklist for interviews and focus groups

**Table 2:** Topic list including interview topics for patients and caregivers

Topics	Questions
General	How did you^a^ experience the stay at the rehabilitation center? [Topics: positive/negative experiences, needs and wishes]
Admission	How did you experience the process of admission to the rehabilitation center? [Topics: day of admission, information provided on day of admission]
Stay	How did you experience being a resident on the Parkinson ward? [Topics: physical environment, activities, contact with peers, food, information provided]
Nursing/medical care	How did you experience nursing and medical care? [Topics: communication, contact, expertise regarding PD, medication administration timing/medication pump, waiting for help]
Therapies	How did you experience therapies? (specifying physiotherapy/ occupational therapy/speech therapy) [Topics: content of program: quantity/usefulness of therapies, needs and wishes, contact with therapists, experiences with group therapy]
Goal setting	Were you aware of your rehabilitation goals? If so: How were you involved in establishing rehabilitation goals? [Topics: multidisciplinary care plan, possibilities to express own wishes and goals, evaluation of goal setting, autonomy]
Outcome of rehabilitation	How do you experience the outcomes of the program?

^a^In caregiver interviews: e.g., ‘your husband/your wife’ [as is appropriate]

**Table 3:** Demographic and clinical characteristics of participants

#	Type	Sex	Age (years)	Diagnosis	Living status	Hoehn & Yahr stage	Years since diagnosis	Caregiver: Relation to patient	SNF	Interviewer
1	P	M	85	MSA	Alone	IV	1	–	1	1*
2	P	M	77	IPD	Alone	III	11	–	1	1*
3	P	F	72	IPD	With spouse	IV	24	–	2	2**
4	P	F	63	IPD	Alone	IV	7	–	2	2**
5	P	F	69	PSP	With spouse	IV	3	–	2	2**
6	P	F	80	IPD	Alone	IV	10	–	2	2**
7	P	F	76	IPD	With spouse	IV	10	–	1	3***
8	P	F	84	IPD	Alone	III	4	–	2	3***
9	P	M	75	IPD	With spouse	III	10	–	1	3***
1	C	M	?	–	With spouse	–	–	Spouse	2	2**
2	C	M	79	–	With spouse	–	–	Spouse	2	2**
3	C	M	73	–	With spouse	–	–	Spouse	2	2**
4	C	F	49	–	Alone	–	–	Daughter	2	3***
5	C	F	58	–	With spouse	–	–	Spouse	1	3***
6	C	M	77	–	With spouse	–	–	Spouse	1	3***

*P* patient, *C* caregiver, *M* male, *F* female, *MSA* multiple system atrophy (MSA), *IPD* idiopathic Parkinson’s disease, *PSP* progressive supranuclear palsy (PSP), *SNF* skilled nursing facility, *?* missing data

^*^Interviewer number 1: sex: male, age: 70, occupation: medical doctor

^**^Interviewer number 2: sex: female, age: 58 years, occupation: nurse practitioner

^***^Interviewer number 3: sex: female, age: 29 years, occupation: physician elderly care medicine, resident

**Table 4:** Themes and subthemes

Themes	Subthemes
I. Autonomy	1. Dependence on help and having to wait
	2. Supervision during rehabilitation
	3. Accessibility of the physical environment
	4. Regaining independence during rehabilitation
II. Sharing information	1. A desire to share personal stories
	2. Making sense of the rehabilitation environment
	3. Interprofessional communication
III. Contact with others	1. Peer contact: positive and negative aspects
	2. Staff members showing empathy
	3. Valuing expertise of staff
